# Subject-Specific Anxiety and Pre-Matriculation Study Preparation Among Incoming Medical Students: A Two-Cohort Analysis

**DOI:** 10.51894/001c.164391

**Published:** 2026-08-01

**Authors:** Vijayashree Jambunathan, Martha Faner, Raquel Ritchie, Sarah E. Tilden, Carolina Restini, John Taylor

**Affiliations:** 1 College of Osteopathic Medicine, Michigan State University

## 43

### Introduction

The transition into medical school is frequently associated with anxiety. Some students choose to prepare for medical school by previewing material prior to matriculation, but little research has examined how anxiety relates to voluntary preparation behaviors. Understanding these relationships may clarify how students approach early academic challenges and inform institutional support strategies.

### Objectives

This study aimed to examine the relationship between subject-specific anxiety and pre-matriculation previewing behaviors among incoming medical students. We hypothesized that students who previewed course material would report lower anxiety. Among students who did not preview, we hypothesized that anxiety would be associated with a greater retrospective desire to have done so.

### Methods

Anonymous survey data were collected from two consecutive cohorts of incoming medical students during orientation (Cohort A: n=173; Cohort B: n=129; IRB STUDY00010320). A follow-up survey was completed one year later by a subset of Cohort A students (n=72) to assess changes in attitudes and study behaviors. Students reported subject-specific anxiety and whether they previewed course material. Among non-previewers, an additional question assessed retrospective desire for previewing. Associations were evaluated using Fisher’s exact, chi-square, odds ratios (OR), and z-tests to compare ORs between cohorts.

### Results

In Cohort B, students reporting subject-specific anxiety had significantly lower odds of having previewed course material compared to those without anxiety (OR 0.11 [0.04-0.30], *p* < 0.001). A similar trend was observed in Cohort A though it did not reach statistical significance (OR 0.36 [0.13-1.01], *p* > 0.05). Contrary to our second hypothesis, anxiety was not significantly associated with an increased retrospective desire to preview among non-previewers in either cohort. At one-year follow-up, anxiety was not significantly associated with a desire to spend more time previewing content prior to matriculation.

### Discussion

Across two cohorts, subject-specific anxiety was associated with lower odds of having previewed course material prior to matriculation. However, among students who did not preview, anxiety did not predict a greater desire to have done so. This suggests motivational or logistical factors beyond anxiety may influence previewing behaviors. Future prospective longitudinal studies are needed to better understand what drives pre-matriculation preparation decisions and inform student support initiatives.

